# Structure–Property Relationship in Cationic Surfactant/Hydroxypropyl Methylcellulose Hydrogels and Cryogels: Role of Headgroup and Counterion Dissociation

**DOI:** 10.1021/acsomega.5c07745

**Published:** 2025-11-12

**Authors:** Victor B. Astuto, Rodrigo Fernandes, Rosangela Itri, Denise F. S. Petri

**Affiliations:** a Fundamental Chemistry Department, Institute of Chemistry, 28133 University of São Paulo, Av. Prof. Lineu Prestes 748, São Paulo 05508-000, Brazil; b Institute of Physics, 28133 University of São Paulo, São Paulo 05508-090, Brazil; c Department of Life and Environmental Sciences, Università Politecnica delle Marche, via Brecce Bianche, Ancona 60131, Italy

## Abstract

Understanding the factors governing the rheological properties of hydrogels and the mechanical properties of the corresponding cryogels obtained by freeze-drying is crucial for diverse applications. In this study, we investigated how incorporating three cationic surfactants, namely, cetyltrimethylammonium bromide (CTAB), cetyltrimethylammonium chloride (CTAC), and cetylpyridinium chloride (CPC), each at 20 mM, affects the rheological behavior of hydroxypropyl methylcellulose (HPMC) aqueous solutions (30 g L^–1^) and the mechanical properties of the resulting cryogels. Among the systems studied, CTAC-containing hydrogels showed the highest storage modulus of *G*′ and the longest relaxation time (τ = 0.685 s), indicating a denser network favored by enhanced counterion dissociation. For comparison, pure HPMC hydrogels presented τ = 0.167 s. Small-angle X-ray scattering (SAXS) data indicated that CTAC micelles possessed the largest intermicellar spacing (238 Å), a result attributable to high ion dissociation, which enhances the electrostatic repulsion among micelles. Cryogels derived from systems containing HPMC and CTAC exhibited a Young’s modulus (*E*) of ∼600 kPa, nearly 3-fold higher than that obtained for pure HPMC cryogels (228 kPa). In contrast, HPMC solutions containing CPC showed intermediate values for τ (0.461 s) and *E* (∼176 kPa), reflecting the lower degree of ion dissociation. The weak dissociation of bromide ions resulted in solutions of HPMC and CTAB with the shortest relaxation time (τ = 0.305 s); these systems crystallized upon freezing, impairing interactions with HPMC chains and leading to brittle cryogels. These findings demonstrated, for the first time, a direct correlation between *G*′ values of HPMC and ionic surfactant solutions and the *E* values of the resulting cryogels; surfactants with higher ion dissociation led to higher *G*′ and *E* values.

## Graphical abstract

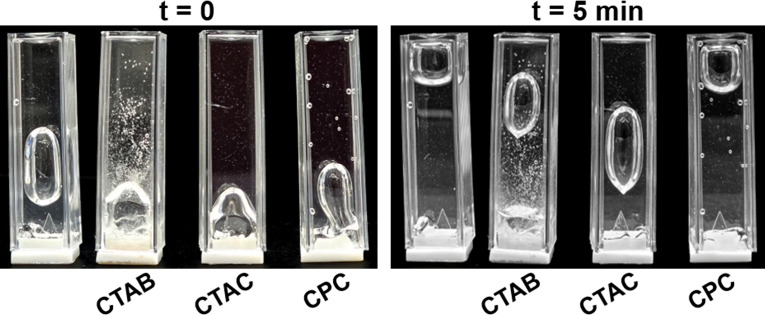

## 1 Introduction

Three-dimensional porous structures are of interest due to their low density and high surface area. These unique properties open possibilities for applications in filtration/adsorption processes,^1^ drug delivery systems or the release of biologically relevant molecules,^2^ energy storage,^3^ and stretching sensors.^4^ Among the various types of porous structures, aerogels, cryogels, and xerogels stand out, all of which share a common starting point: precursor gels.^5^ Typically, the production of aerogels involves replacing the solvent in the precursor gel with CO_2_ under supercritical conditions. When the system pressure is reduced, CO_2_ changes from the liquid to the gaseous phase, leaving behind mesopores (2 to 50 nm) in the resulting structure.^5^ Cryogels are prepared by freeze-drying.^6,7^ When the solvent is water, cryogel formation involves the exclusion of polymer chains and solutes from growing ice crystals during freezing, leading to their accumulation in the intercrystalline regions. This localized concentration drives network formation and ultimately defines the porous structure after ice sublimation. The cryoconcentrated regions become solid walls of the cryogel. Xerogels are obtained through simple evaporation of the solvent, resulting in collapsed pores. The use of polysaccharide-based gels for the fabrication of aerogels and cryogels has been extensively explored due to their abundance, structural diversity, biocompatibility, and biodegradability.^7–9^

Despite the growing interest in cryogels and aerogels, there are few systematic investigations elucidating the specific physicochemical parameters and molecular interactions that govern the mechanical behavior of precursor gels and their corresponding cryogels or aerogels, which are critical for optimizing the performance in targeted applications. As a general trend, when the formulation reinforces network stiffness, precursor gels exhibit a storage modulus (*G*′) greater than the loss modulus (*G*″), and the resulting cryogels or aerogels show an enhanced compressive modulus (*E*). For instance, TEMPO-oxidized nanofibrillated cellulose (CNF) suspensions with *G*′ > *G*″ allowed predicting the corresponding aerogel properties with respect to xylan content and surface bound water and, therefore, to the mechanical properties.^10^ A linear relationship between the storage modulus *G*′ and the compressive modulus *E* was observed in systems with systematically increased concentrations of oxidized CNFs, both in the presence and absence of NaCl.^11^ The *G*′ values of the poly­(vinyl alcohol)/cellulose nanocrystal (PVA/CNC) hydrogels, as well as the *E* values of the corresponding cryogels, were found to increase with CNC content.^12^ Increasing the number of freeze–thaw cycles of polyphenol-loaded xanthan gum/poly­(vinyl alcohol) hydrogels promoted the increase of both *G*′ and *E* values.^13^ Shorter diglycidyl ethers led to cross-linked chitosan networks with higher *G*′ and *E* values.^14^ Adding fructose to collagen led to stiffer cross-linked hydrogels and cryogels.^15^

Hydroxypropyl methylcellulose (HPMC) is a nonionic, water-soluble cellulose ether that forms viscous solutions upon dissolution in water. Its thickening behavior depends on several factors, including molecular weight, degree of substitution (DS), molar substitution (MS), concentration, and temperature.^16,17^ In a previous study, we reported the increase in both the storage modulus (*G*′) and compressive modulus (*E*) of HPMC systems with increasing concentrations of the anionic surfactants sodium dodecyl sulfate (SDS) and bis­(2-ethylhexyl) sodium sulfosuccinate (AOT).^18^ This enhancement was attributed to favorable interactions between the charged head groups of the surfactants and HPMC chains, facilitating multipoint attachment of micelles to HPMC residues.^18,19^ These findings demonstrated that micelle addition alone can significantly stiffen both hydrogels and cryogels without altering polymer concentration.^18,19^ However, limited information is available in the literature on how the surfactant headgroup and counterion influence the mechanical properties of such systems. In this study, we selected three cationic surfactants, namely, cetyltrimethylammonium bromide (CTAB), cetyltrimethylammonium chloride (CTAC), and cetylpyridinium chloride (CPC), commonly used in commercial hair conditioners and mouthwashes. These surfactants share a C16 alkyl chain but differ in their headgroups and counterions. We systematically investigated how these structural variations affect the storage modulus (*G*′) of HPMC precursor hydrogels (30 g L^–1^) and the compressive modulus (*E*) of the resulting cryogels. Our goal was to expand the range of surfactants capable of reinforcing hydrogel and cryogel matrices and to deepen the understanding of how the surfactant structure governs interactions with HPMC, ultimately influencing their mechanical performance. To the best of our knowledge, there is scarce information in the literature about the effect of counterion dissociation of ionic surfactants on the correlation between *G*′ values of hydrogel precursors and the *E* values of the resulting cryogels.

## 2 Materials and Methods

### 2.1 Materials

Hydroxypropyl methylcellulose J12MS (USP HPMC 1828, DS 1.5, MS 0.75) was kindly supplied by The Dow Chemical Company (Brazil); the viscometric average molar mass (*M*_v_) of 3.46 × 10^5^ g/mol was determined by capillary viscometry.^19^ Cetyltrimethylammonium bromide (CTAB, H5882, 364.45 g/mol, purity >98%), cetyltrimethylammonium chloride (CTAC, 52366, 320.00 g/mol, purity >98%), and cetylpyridinium chloride (CPC, C9002, 358.00 g/mol, purity >95%) were provided by Sigma-Aldrich and used as received. Scheme 1 presents the chemical structures of the repetitive unit of HPMC, CPC, CTAC, and CTAB. Milli-Q water was used for the solution preparation.

**Scheme 1:**
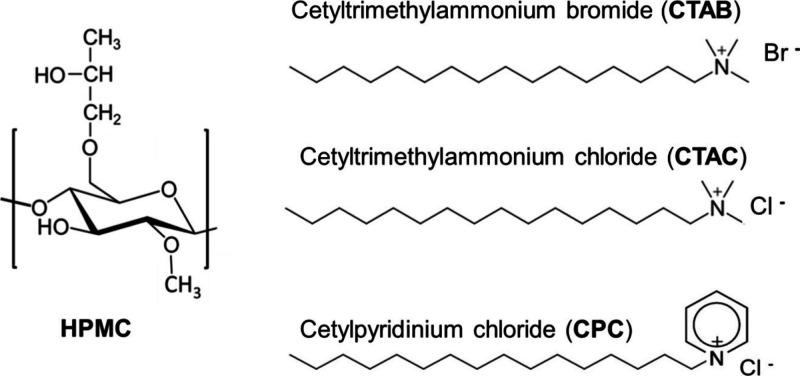
. Representation of the Chemical Structures of HPMC, CTAB, CTAC, and CPC

### 2.2 Preparation of HPMC Precursor Hydrogels and Cryogels

Aqueous solutions of HPMC were prepared at 30 g L^–1^ and homogenized for 2 h.^18^ After that, the systems were kept in a refrigerator at 7 °C overnight to improve the solubility of HPMC in water and to eliminate bubbles.^18^ In this work, the term “precursor hydrogel” refers to the concentrated polymer solution, which still flowed slowly when the vials were tilted.

Aqueous solutions of CTAB, CTAC, and CPS were prepared at 20 mM under magnetic stirring at 24 ± 1 °C. At this temperature, the critical micelle concentration (cmc) values of CTAB,^20^ CTAC,^21^ and CPC^22^ in water are ∼1.0 mM for all of them. Then, HPMC powder was added to the surfactant solution, so that the final concentration of HPMC was always 30 g/L. The precursor hydrogels were coded as HPMC (without surfactant), HPMC-CTAC20, HPMC-CTAB20, and HPMC-CPC20. In the case of CTAB, hydrogels containing 5 mM CTAB (HPMC-CTAB5) were also prepared. The precursor hydrogels were transferred to acrylic cylindrical molds and frozen for 24 h in a standard freezer at −33 °C. Then, they were freeze-dried under vacuum (∼0.2 mbar), −51 °C, for 12 h. Scheme 2 depicts the preparation of the precursor hydrogels and the corresponding cryogels.

**Scheme 2:**
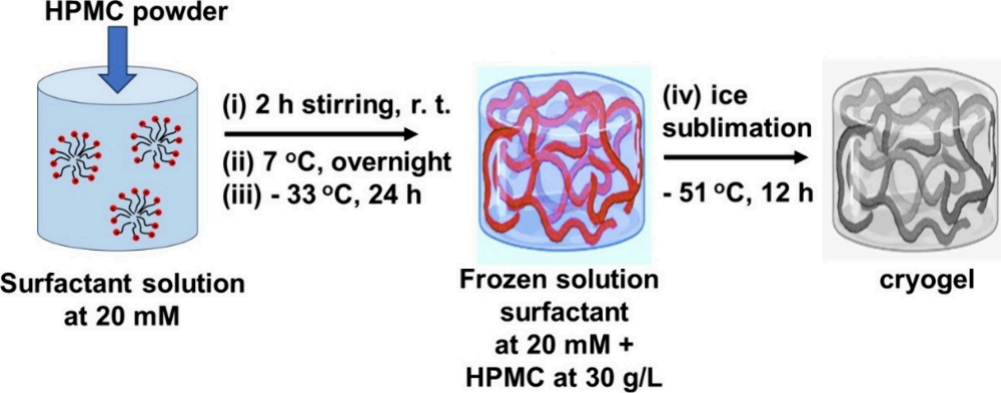
– Experimental Steps for the Preparation of the Precursor Hydrogels and Corresponding Cryogels^a^

a (i) Addition of HPMC (powder) to aqueous solutions of CTAB, CTAC, or CPS at 20 mM, magnetic stirring at 24 ± 1 °C, for 2 h. (ii) The systems were kept in the refrigerator at 7 °C overnight. (iii) After pouring into molds, the systems were frozen at −33 °C for 24 h. (iv) Sublimation of ice crystals at 0.2 mbar and −51 °C for 12 h.

### 2.3 Characterization of Hydrogel Precursors

Rheological tests were performed using a stress-controlled MCR 501 rheometer by Anton Paar at (25.0 ± 0.5) °C under a dry nitrogen atmosphere. Cone and plate geometry was used with an angle of 0.992°, a diameter of 50 mm, and a gap size of 0.101 mm. Strain sweep tests were carried out for all hydrogels to define the linear viscoelasticity region. The pure HPMC, HPMC-CTAC20, HPMC-CTAB20, and HPMC-CPC20 hydrogels were tested at frequencies of 1 and 10 Hz, measuring the storage (*G*′) and loss (*G*″) moduli as a function of the strain amplitude. In order to evaluate the *G*′ and *G*″ moduli of precursor hydrogels, dynamic frequency sweep tests were performed by varying the frequency from 0.1 to 10 Hz, keeping the strain amplitude at 0.5%. All dynamic frequency sweep tests were performed at 0.5% strain because it corresponded to the linear viscoelastic region (Figure S1). The samples were measured at least three times. The differences among the replicates were ≤2%.

Viscosity flow curves (viscosity as a function of shear rate) were obtained for pure HPMC, HPMC-CTAC20, HPMC-CTAB20, and HPMC-CPC20 hydrogels using the same cone-and-plate setup employed in the oscillatory tests at a constant temperature of (25.0 ± 0.5) °C. The viscosities (η) of pure HPMC, HPMC-CTAC20, HPMC-CTAB20, and HPMC-CPC20 hydrogels were measured at a constant shear rate of 1 s^–1^ under a cooling ramp from +50 to −5 °C at a rate of 1 °C/min. Rheological analysis during cooling was essential to assess the behavior of the samples during freezing, a critical step prior to lyophilization. The *G*′ and *G*″ of pure HPMC, HPMC-CTAC20, HPMC-CTAB20, and HPMC-CPC20 hydrogels were also measured at a frequency of 10 Hz under a cooling ramp from +50 °C to −10 °C (HPMC and HPMC-CTAB20) or +50 °C to −5 °C (HPMC-CTAC20 and HPMC-CPC20) at a rate of 1 °C/min. Due to the crystallization behavior of CTAB under cooling,^23^ η was measured at a constant shear rate of 1 s^–1^ under a cooling ramp from +50 °C to −5 °C at a rate of 1 °C/min for hydrogels containing 5 mM, 10 mM, 20 mM, and 40 mM CTAB, corresponding to the systems HPMC-CTAB5, HPMC-CTAB10, HPMC-CTAB20, and HPMC-CTAB40, respectively.

Small angle X-ray scattering (SAXS) experiments were performed using a SAXS setup with a GeniX 3D source (Xenox) and a Pilatus 300k detector at the Crystallography Laboratory, Institute of Physics from the University of São Paulo (USP, Brazil). Samples were composed of CTAB, CTAC, and CPC aqueous solutions at 20 mM, and HPMC (30 g/L), HPMC-CTAC20, HPMC-CTAB20, and HPMC-CPC20 hydrogels at (25 ± 1) °C. All samples were placed in a glass capillary (2.0 mm diameter) and measured over 60 min. The SAXS data were averaged over 5 consecutive runs. The scattering vector modulus *q* = 4π sin θ_SAXS_/λ, with 2θ_SAXS_ being the scattering angle and λ being the X-ray wavelength of 1.548 Å, ranged from 0.026 to 0.26 Å^–1^. The scattering intensity, *I*(*q*), can be described as a product of a structure factor, *S*(*q*), and a form factor, *P*(*q*),^24,25^ such that

I(q)=kP(q)S(q) 1

where *k* depends on the particle number density, the electron density contrast between the scattering particle and the medium and the scattering particle volume. For systems with small polydispersity (∼20%), the deviation in eq 1 corresponds to a diffuse background scattering that was accounted for in our data treatment.

In this work, we make use of the Genfit software^26^ to analyze the scattering data by fitting a model (eq 1) to the experimental data. In the case of CTAB, CTAC, and CPC at 20 mM, the *P*(*q*) form factor is represented by a prolate ellipsoid made up of two shells of distinct electron densities ρ with respect to the solvent electron density (in our case, ρ_w_ = 0.333 e/Å^3^ for aqueous solution), and the *S*(*q*) structure factor assumes that surface-charged micelles interact through a screened electrostatic potential, as described elsewhere.^27^ The prolate micelle, with anisometry ν, is represented by a hydrophobic paraffinic core with electron density ρ_par_ = 0.275 e/Å^3^; the shortest semiaxis length is associated with the parameter *r*_par_, and the longest semiaxis length to ν*r*_par_. Such a hydrophobic core is surrounded by a polar shell of thickness σ and electron density ρ_shell_, which includes the polar head groups and hydration water.

For the HPMC/CTAB, HPMC/CTAC, and HPMC/CPC systems, a necklace model^28–30^ was used to fit the SAXS data. This model assumes that the HPMC/surfactant complex consists of a string of micelle-like aggregates distributed along the polymeric chain and suspended in random orientations in solution. P­(q) in eq 1 is modeled as a prolate ellipsoid-like micelle described above. In terms of S­(*q*) (eq 1), one can derive its asymptotic behavior at small *q* by assuming that in a df -dimensional space, the number *N*(*r*) of individual scatters within a sphere of radius *r* is given by *N*(*r*) = (*r*/*R*)^df^, where *R* is an effective sphere micellar radius with an equal inner hydrocarbon-core volume of the ellipsoidal micelle, surrounded by a polar shell of thickness σ (*R* = ν ^1/3^ *r*_par_ + σ). The physical meaning of df can be seen as a fractal dimension of the micellar clusters. Furthermore, the micelle–micelle correlation will have a finite range ξ associated with the persistence length for polymers.

### 2.4 Characterization of the Cryogels

The mean apparent density (ρ_app_) of the cryogels was determined in triplicate at 23 ± 1 °C and relative air humidity of 65 ± 10% by the ratio between their masses (*m*_pol_) determined in an analytical balance and their volumes measured by a caliper. Scanning electron microscopy (SEM) analyses were performed for cryogels coated with a thin gold layer using a JEOL Neoscope JCM-5000 microscope, operating at a voltage of 10 kV. Dynamic mechanical analysis (DMA, TA Q800) was performed to investigate the mechanical properties of the cryogels at (25 ± 1) °C in air (60% relative humidity). At least five samples, each 10 mm thick and 10 mm in diameter, were analyzed. DMA was performed in compression mode using a uniaxial compressive force ranging from 1 to 18 N, applied at a constant rate of 1 N·min^–1^, with a preload force of 0.001 N. The FTIR-ATR vibrational spectra of all cryogels were obtained in the spectral range of 600 to 4000 cm^–1^ using Frontier PerkinElmer equipment, a ZnSe crystal, and an accumulation of 32 scans (4 cm^–1^ resolution). Analysis of variance (ANOVA) was carried out using Excel to determine whether the observed differences among data groups were statistically significant, with significance defined at *p* < 0.05.

## 3 Results and Discussion

### 3.1 Rheological Properties and SAXS Measurements of Hydrogels at 25.0 °C

Figure 1a–d shows the storage modulus *G*′ and loss modulus *G*″ as a function of the frequency measured for HPMC, HPMC-CTAC20, HPMC-CTAB20, and HPMC-CPC20 hydrogels at 25.0 ± 0.5 °C. At low frequencies, the average *G*″ values exceeded the average *G*′ values, whereas at higher frequencies, *G*′ values became dominant, indicating that all systems exhibited viscoelastic fluid behavior. The addition of surfactants to HPMC hydrogels led to a reduction in both the crossover modulus (where *G*′ = *G*″) and the corresponding frequency. The most pronounced effect was observed with CTAC, where the crossover modulus decreased from 151 to 125 Pa and the frequency dropped from 5.97 to 1.46 Hz. It means that the relaxation time (τ), which is the reciprocal of frequency, increased from 0.167 to 0.685 s due to the increase of entanglements. In contrast, the smallest effect was in the presence of CTAB, with a decrease from 151 to 133 Pa and a frequency shift from 5.97 to 3.28 Hz. In this case, τ increased only from 0.167 to 0.305 s. These results suggest that HPMC chains form more junction points in the presence of CTAC micelles compared to CTAB, resulting in a stiffer network. This behavior is likely due to the quaternary ammonium groups on the micelle surface, which can interact with hydroxy groups from different HPMC chains, thereby reinforcing the structure. However, in the case of CTAB, 77% of Br^–^ ions are strongly associated with the positively charged micelle surface,^31^ reducing the number of effective interactions with HPMC residues. In comparison, only 58% and 51% of chloride ions are associated with the CTA^+^ or CP^+^ micelle surface, respectively.^32^ Bromide ions are larger and more polarizable than chloride ions, making their dissociation less favorable.

**Figure 1:**
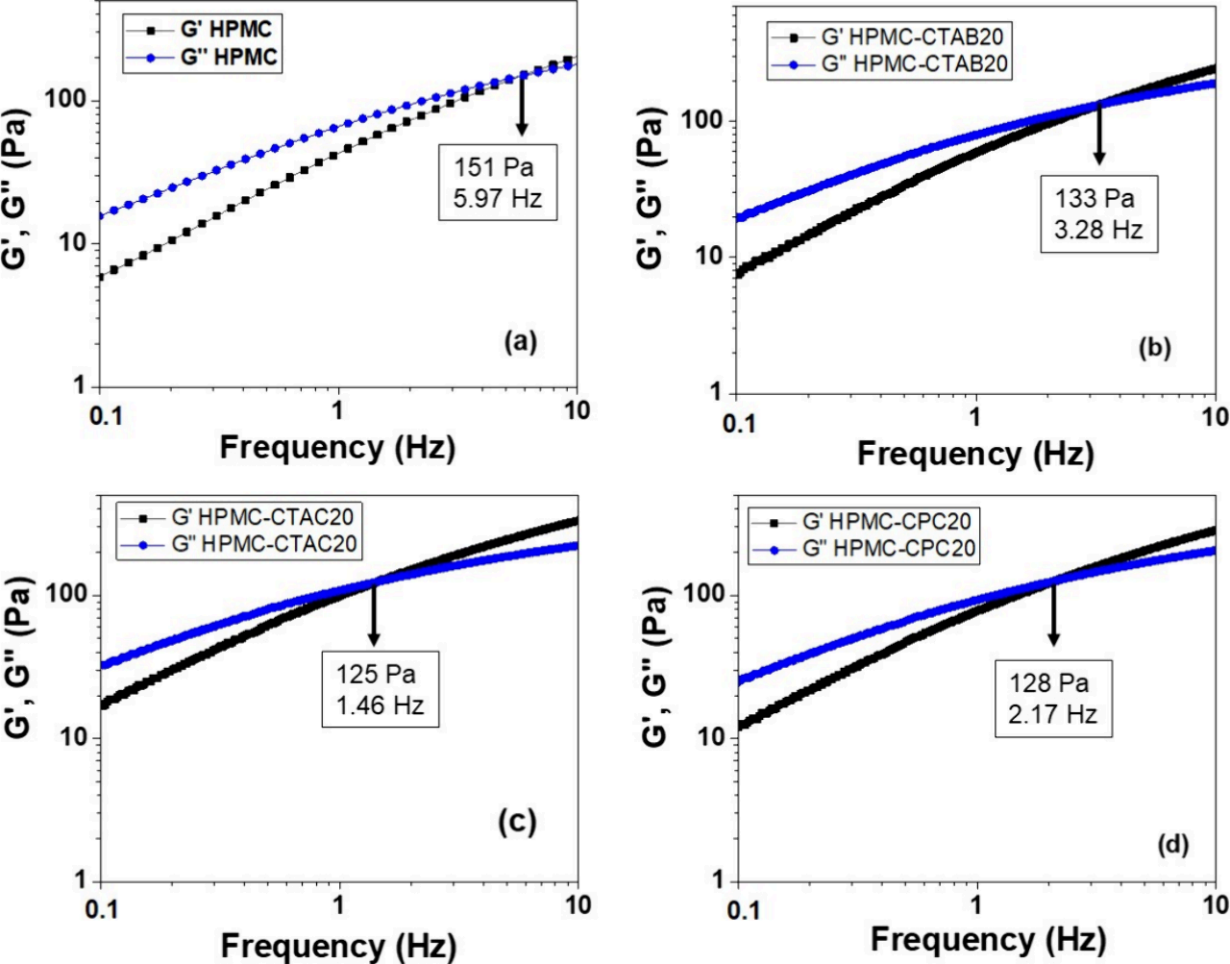
Average storage modulus *G*′ and loss modulus *G*″ as a function of frequency determined for (a) pure HPMC (30 g/L), (b) HPMC-CTAB20, (c) HPMC-CTAC20, and (d) HPMC-CPC20 systems at 25.0 ± 0.5 °C.

In order to gain insight about the interactions between HPMC and the micelles of the different cationic surfactants, SAXS measurements were performed at (25 ± 1) °C for CTAB, CTAC, and CPC aqueous solutions at 20 mM and for HPMC (30 g/L), HPMC-CTAB20, HPMC-CTAC20, and HPMC/CPC20 hydrogels. The Supporting Information shows the typical SAXS curves obtained for CTAB, CTAC, and CPC aqueous solutions at 20 mM, along with the best fitting curves. The Supporting Information (Figure S3) summarizes the structural parameters extracted from these fittings, which were consistent with literature values.^33–35^ The micelles exhibited anisotropy values between 2.5 and 3.2, consistent with prolate ellipsoidal shapes.^36,37^ The *r*_par_ values ranged from 20.8 ± 2.4 Å to 25.8 ± 4.4 Å, which are reasonable values for C16 alkyl chains.^38^ The thickness of the polar shells (σ) and the corresponding electron density (ρ_shell_) were comparable for all three surfactants.

Figure 2a shows the experimental SAXS curves determined for pure HPMC precursor gel (30 g/L) (black), HPMC-CTAB20 (blue), (c) HPMC-CTAC20 (green), and (d) HPMC-CPC20 (red) hydrogels. The SAXS curves of pure HPMC hydrogels were fitted using a wormlike model with a finite cross-section, yielding a Kuhn length of 98.6 ± 8.5 Å, a contour length of 153.4 ± 3.7 Å, and a cross-sectional radius of 5.02 ± 0.27 Å. The magnitude of the Kuhn length indicates that HPMC chains within the hydrogel are flexible and probably arranged as random coils.^39^

**Figure 2:**
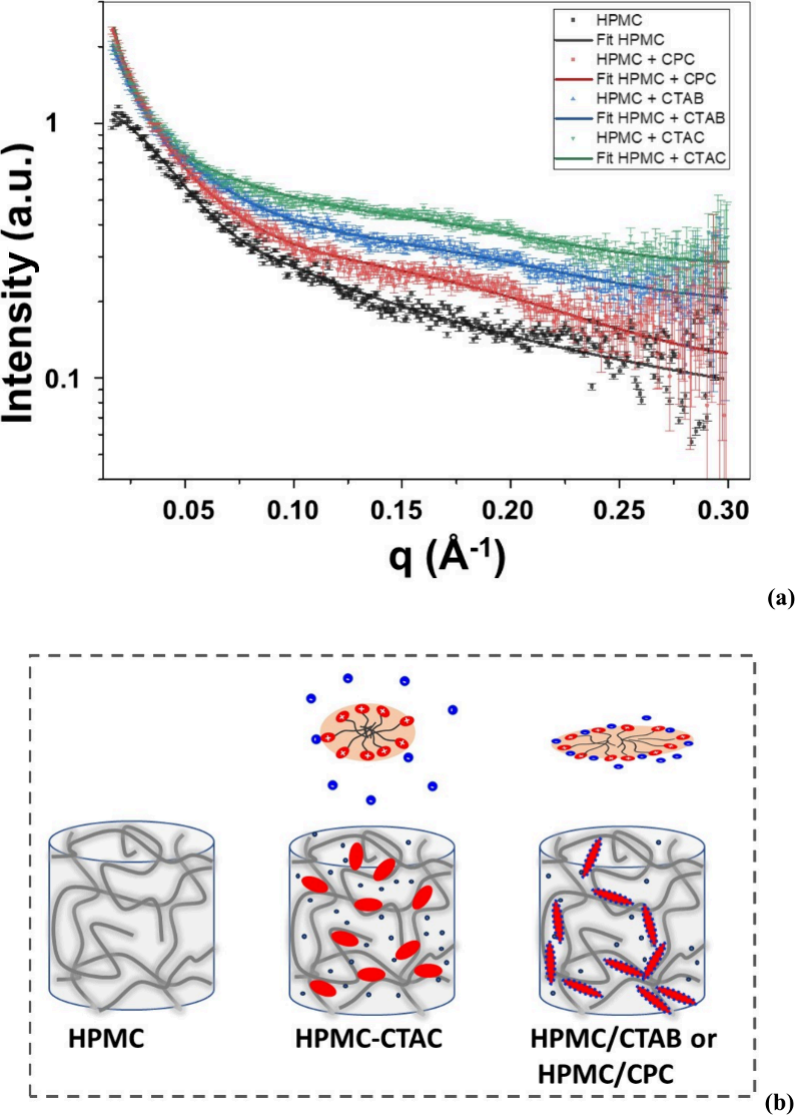
(a) Experimental SAXS curves determined for pure HPMC precursor gel (30 g/L) (black), HPMC-CTAB20 (blue), (c) HPMC-CTAC20 (green), and (d) HPMC-CPC20 (red) hydrogels, along with the fitting curves. (b) Schematic representation of HPMC hydrogels in the presence of CTAC, CTAB, or CPC micelles, where the blue spheres represent the counterions.

All experimental SAXS data determined for the systems containing surfactants fitted well to the string-of-pearls or necklace model,^33^ where the polymer chains interact with the charged outer layer of the micelles. Table 1 shows the micelle anisometry (ν), mean thickness of the polar shell (σ), mean electron density of the shell (ρ_shell_), and mean intermicellar distance (*d*_int_) in the HPMC hydrogel.

**Table 1:** Anisometry (ν), Mean Thickness of the Polar Shell (σ), Mean Electron Density of the Shell (ρ_shell_), and Mean Intermicellar Distance (*d*_int_) of CTAB, CTAC, and CPC Micelles in Water and in HPMC Hydrogels at (25 ± 1) °C

system	ν	σ (Å)	ρ_shell_ (e/Å^3^)	*d*_int_ (Å)
CTAB	2.5	5.2 ± 1.4	0.35 ± 0.03	
CTAC	3.2	5.1 ± 1.3	0.343 ± 0.004	
CPC	2.5	5.3 ± 1.6	0.35 ± 0.01	
HPMC-CTAB20	3.9	6.7 ± 2.1	0.375 ± 0.007	97 ± 5
HPMC-CTAC20	3.4	5.8 ± 3.8	0.358 ± 0.004	238 ± 10
HPMC-CPC20	4.3	5.8 ± 3.8	0.35 ± 0.01	104 ± 6

Compared with the pure micelles, changes in the σ and ρ_shell_ values were not significant. However, the anisotropy of CTAB and CPC micelles increased markedly, whereas the increase was less pronounced for CTAC. These findings indicate that the CTAB and CPC micelles in the HPMC gels are more elongated than in water. The *d*_int_ values, representing the center-to-center spacing between micelles, were smaller for CTAB and CPC than for CTAC. For CTAB, the reduced micellar spacing is attributed to the lower dissociation tendency of bromide ions, which remained more tightly bound to the micelle surface compared to chloride ions. This stronger bromide pairing led to weaker electrostatic repulsion between adjacent micelles, allowing them to be closer to each other. In the case of CPC, the planar geometry of the pyridinium headgroup promotes stronger association with chloride ions than the more sterically hindered quaternary ammonium group in CTAC. Density functional theory (DFT) calculations support this, showing that the N–Br distance in CTAB is 3.72 Å, while the N–Cl distance in CPC is shorter, at 3.04 Å.^40^ Consequently, CTAC exhibited the largest *d*_int_ values, indicating greater chloride ion dissociation and, therefore, stronger intermicellar electrostatic repulsion.^37^ In this scenario, the cationic charges on the surface of CTAC micelles are more accessible for interaction with HPMC chains, as they are less screened by counterions compared with those on CTAB or CPC micelles. This greater availability facilitates a higher number of contact points between CTAC micelles and the HPMC network, resulting in the lowest crossover frequency observed in Figure 1c. Figure 2b depicts the possible interactions between the CTAC and HPMC chains and CTAB or CPC and HPMC chains in the hydrogels.

### 3.2 Rheological Properties of Hydrogels from +50 °C to −5 °C

Considering that the precursor hydrogels are transformed into cryogels through freeze-drying, it is essential to evaluate the structural changes that may occur during the cooling process. The measurements were carried out under a controlled cooling ramp from +50 to −5 °C at a rate of 1 °C/min and at a fixed frequency of 10 Hz. At this frequency and +25 °C, the *G*′ values of HPMC (30 g/L), HPMC-CTAB20, HPMC-CTAC20, and HPMC-CPC20 systems were higher than their *G*″ values, indicating a predominantly “solid-like” behavior under these conditions (Figure 1). The temperature scan aimed to evaluate the influence of temperature on this viscoelastic behavior.

Figure 3a–d displays the *G*′ and *G*″ moduli as a function of temperature for HPMC, HPMC-CTAC20, HPMC-CTAB20, and HPMC-CPC20 hydrogels, respectively. This frequency was selected because, at +25 °C, the storage modulus (*G*′) was higher than the loss modulus (*G*″), as shown in. Except for the pure HPMC hydrogel (Figure 3a), all other systems exhibited *G*′ values higher than *G*″ and remained approximately constant throughout the temperature range of +50 °C to +5 °C. This behavior suggests that cooling promotes hydrogen bonding among HPMC chains,^41,42^ which would typically increase both *G*′ and *G*″. However, this effect appears to be counterbalanced by the lubricating action of micelles, which helps maintain the moduli nearly constant. For the pure HPMC hydrogel, *G*′ = *G*″ at 47.5 °C. Upon further cooling, the increase in *G*′ becomes more pronounced than that in *G*″, likely due to enhanced hydrogen bonding within the polymer network. Below 5 °C, significant increases in both *G*′ and *G*″ are observed in systems containing CTAB and CPC, which are attributed to the crystallization of these surfactants. This effect is more pronounced in CTAB-containing systems, likely due to the weaker dissociation of the bromide counterion. Furthermore, a comparison between CTAC and CPC suggests that the flat pyridinium headgroup of CPC binds chloride ions more strongly than the tetraalkylammonium head of CTAC.^40^ As a result, systems containing CTAC did not exhibit crystallization under similar conditions.

**Figure 3:**
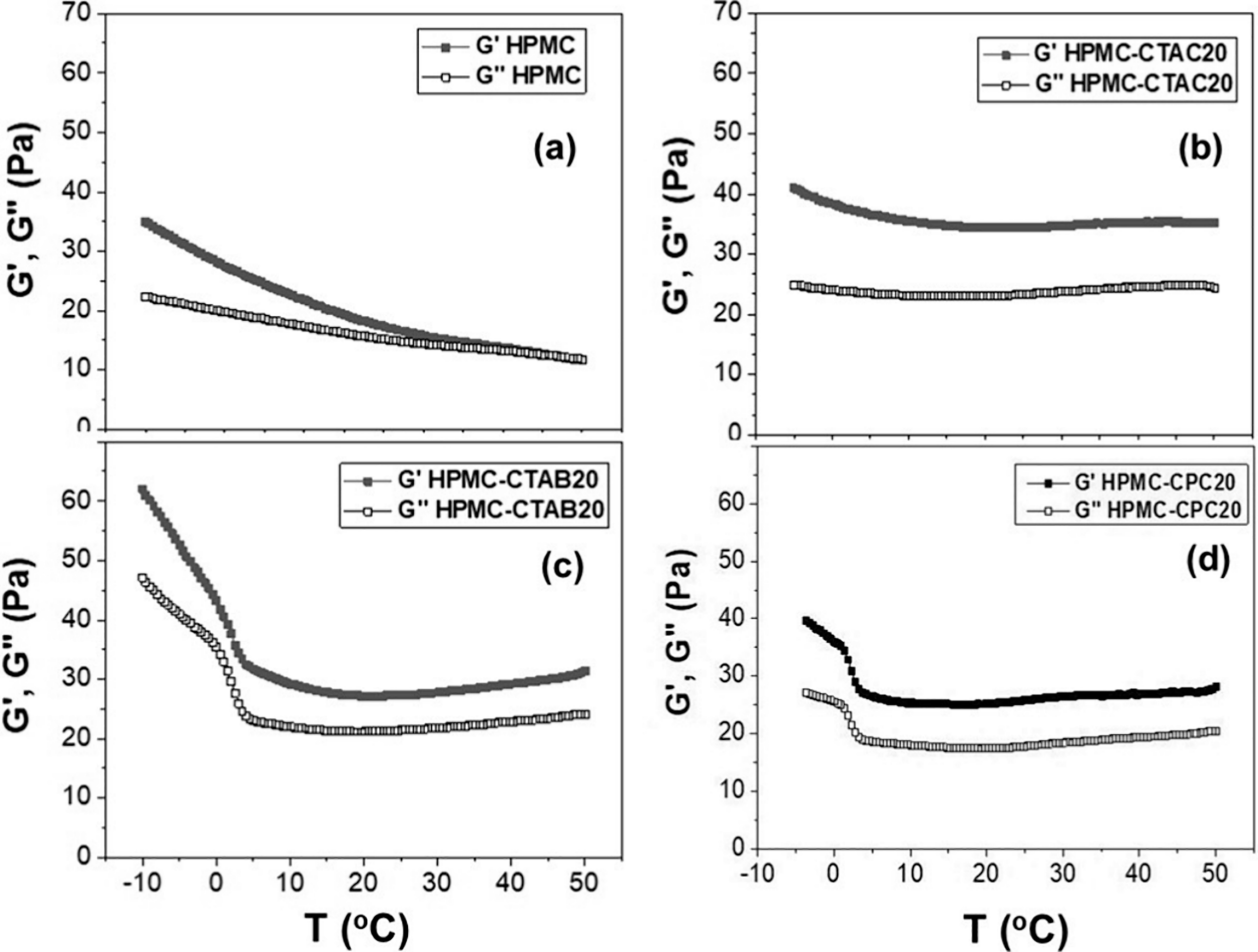
Storage modulus (*G*′) and loss modulus (*G*″) as a function of temperature for (a) HPMC, (b) HPMC-CTAC20, (c) HPMC-CTAB20, and (d) HPMC-CPC20 hydrogels. The measurements were carried out under a controlled cooling ramp from +50 °C to −5 °C at a rate of 1 °C/min and at a fixed frequency of 10 Hz.

The Supporting Information (Figure S4) shows the viscosity flow curves determined for pure HPMC (30 g/L), HPMC-CTAB20, HPMC-CTAC20, and HPMC-CPC20 hydrogels at 25 °C, along with their corresponding fits to the Carreau model. All systems presented shear thinning behavior (*n* < 0.5), and the rotational viscosity (η) values followed the trend HPMC-CTAC20 > HPMC-CTAB20 > HPMC-CPC20 > HPMC hydrogels. Consistent with the oscillatory rheological behavior observed at 25 °C (Figure 1), the pronounced dissociation of chloride ions in CTAC micelles facilitated stronger interactions between the positively charged headgroups and the HPMC hydroxy groups.

Figure 4a presents the rotational viscosity profiles of pure HPMC (30 g/L), HPMC-CTAB20, HPMC-CTAC20, and HPMC-CPC20 hydrogels, measured at a constant shear rate of 1 s^–1^ under a controlled cooling ramp from +50 to −5 °C at a rate of 1 °C/min. Aqueous solutions of HPMC are known to exhibit thermogelation, which typically begins with phase separation near 65 °C, followed by gelation around 75 °C.^43–45^ To avoid the onset of thermogelation and focus solely on the behavior during cooling, the maximum temperature was set at +50 °C. For the pure HPMC hydrogel (black symbol), η values increased exponentially as the temperature decreased (Figure S5), yielding an activation energy (*E*a) for a viscous flow of 25.6 kJ·mol^–1^. This value is consistent with literature data reported for HPMC samples with a degree of substitution (DS) of 1.1, a molar substitution (MS) of 0.11, and a number-average molecular weight (*M*_n_) of 90 kg·mol^–1^, dissolved in water at the same concentration.^43^ In the range from +50 to +35 °C, the systems containing surfactants presented small peaks in the η values, which were attributed to concentration fluctuations due to possible local phase separation. From +35 to +10 °C, the increase of η values of 4.2, 5.0, and 7.0 Pa·s in the presence of CPC, CTAB, and CTAC micelles, respectively, was smaller than that observed for pure HPMC (17.2 Pa·s). This finding indicated that the micelles act as efficient junction points among the HPMC chains, increasing the thermal stability of the hydrogels. Between +50 and +35 °C, the systems containing surfactants exhibited small peaks in viscosity (η), which were attributed to concentration fluctuations possibly caused by local phase separation.

**Figure 4:**
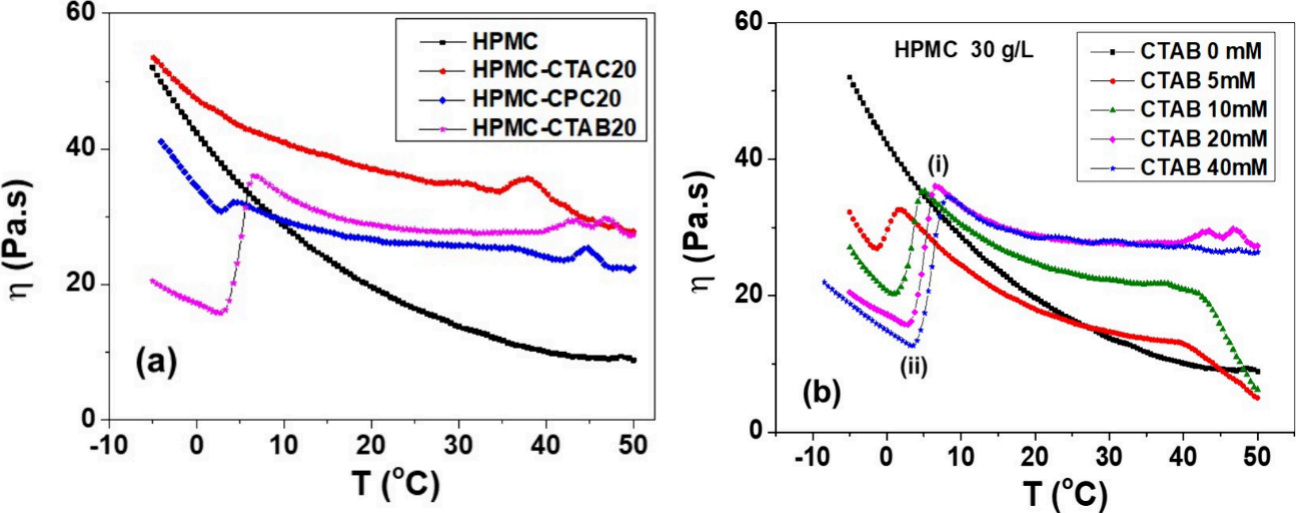
(a) Viscosity values of pure HPMC (30 g/L, black symbol), HPMC-CTAB20 (magenta symbol), HPMC-CTAC20 (red symbol), and HPMC-CPC20 (blue symbol) hydrogels. (b) Viscosity values of pure HPMC (black symbol) and HPMC hydrogels containing 5 mM (red symbol), 10 mM (green symbol), 20 mM (magenta), and 40 mM (blue symbol) CTAB. Measurements at a constant shear rate of 1 s^–1^ under a controlled cooling ramp from +50 to −5 °C at a rate of 1 °C/min.

From +20 °C down to −5 °C, the η measured for the system containing CTAC increased exponentially, yielding an *E*a for a viscous flow of ∼9 kJ·mol^–1^ (Figure S6). It means that the addition of CTAC considerably reduced the *E*a for viscous flow determined for pure HPMC (25.6 kJ·mol^–1^), probably because the micelles reduced the H bonding among the HPMC chains and acted as lubricants. A similar effect was observed for HPMC added to dispersions of cellulose microfibrils^46^ or to an aqueous solution of carboxymethyl cellulose.^47^ In contrast, a maximum was observed in the systems containing CTAB (at 6.5 °C) and CPC (at 4.7 °C), followed by a minimum near 3.0 °C. These features were attributed to the crystallization of the CTAB or CPC surfactants and the onset of ice crystallization, respectively. Upon crystallization, interactions between CTAB or CPC crystals and the HPMC chains weakened, driving the viscosity down to a minimum. At that point (near 3.0 °C), ice began to nucleate; with further cooling, the growing solid fraction caused the viscosity to rise again. Since pure water freezes at 0 °C under 1 atm, it is plausible that CTAB and CPC act as nucleating agents. This effect is more pronounced in CTAB-containing systems than in those prepared with CPC.

To gain further insight into these transitions, HPMC hydrogels were prepared with varying concentrations of CTAB (5, 10, 20, and 40 mM). Figure 4b shows the temperature dependence of viscosity, with the maximum and minimum indicated as points (i) and (ii), respectively. As the CTAB concentration increased, both the maximum and minimum shifted to higher temperatures (Figure S7), supporting the interpretation that these features correspond to CTAB crystallization and the onset of ice crystallization, respectively. Similar behavior was observed for the sol–gel transition temperatures of CTAB, CTAC, and CPC (at 150 mM) hydrogels containing 12-hydroxyoctanodecanoic acid, an organogelator; the transition temperature increased with the surfactant, and it was the highest for CTAB due to its lowest solubilization.^32^

Figure S8 shows the viscosity (η) measurements performed at a constant shear rate of 1 s^–1^ under a cooling–heating cycle (1 °C min^–1^). While pure HPMC hydrogels (30 g L^–1^) exhibited no thermal hysteresis, the melting transitions of ice and CTAB crystals in HPMC-CTAB5, HPMC-CTAB20, and HPMC-CTAB40 systems occurred on average 3 to 5 °C above their respective crystallization temperatures. The presence of CTAB might affect interfacial energies, serving as heterogeneous nucleation sites and shifting crystallization and melting temperatures asymmetrically.^48^ Consequently, the activation energy for nucleation can be reduced in the presence of CTAB, shifting the onset of crystallization to higher temperatures relative to pure systems.^49^ Upon heating, these CTAB-templated nuclei also require increased thermal energy for melting, producing an asymmetrical elevation of melting temperatures and resulting in thermal hysteresis.

### 3.3 Characterization of the Cryogels

The results presented so far demonstrated that cooling pure HPMC and HPMC-CTAC20 hydrogels induced an exponential rise in viscosity, whereas HPMC-CPC20, and especially HPMC-CTAB20, displayed crystallization events under the same conditions.

The precursor hydrogels of pure HPMC, HPMC-CTAC20, HPMC-CTAB20, and HPMC-CPC20 were freeze-dried and subsequently characterized. Figure S9 presents the FTIR-ATR spectra of HPMC, HPMC-CTAB20, HPMC-CTAC20, and HPMC-CPC20 cryogels. All spectra were generally similar and exhibited the characteristic bands of HPMC, including a broad absorption in the 3600–3000 cm^–1^ region corresponding to the O–H stretching vibrations; bands at 2930 cm^–1^ and 2850 cm^–1^, attributed to the asymmetric and symmetric −CH_2_– stretching, respectively; and signals in the 1200–850 cm^–1^ region associated with the C–O and C–C stretching vibrations of the glucopyranose ring.^50^ Additional bands at 1637 cm^–1^, 1463 cm^–1^, and 1378 cm^–1^ were assigned to the O–H bending (δOH) of HPMC or adsorbed water, C–H bending (scissoring or deformation) of −CH_2_– and −CH_3_ groups, and symmetric CH_3_ deformation, respectively. Cryogels containing surfactants displayed more intense and sharper bands at 2930 cm^–1^ and 2850 cm^–1^, reflecting the contribution of the C16 alkyl chains from the incorporated surfactants.

The HPMC-CTAB20 cryogels exhibited significant fragility, complicating their handling and processing. This behavior was attributed to CTAB crystallization, which likely induced phase separation and disrupted the network homogeneity. Consequently, a formulation with reduced CTAB content (HPMC-CTAB5) was selected for freeze-drying and subsequent physicochemical characterization.

SEM images (Figure 5) revealed that all cryogels exhibited the characteristic open porous morphology typical of cryogel structures. Crystalline domains were observed in the HPMC-CTAB cryogels, which is consistent with CTAB crystallization during the freezing process, as indicated in Figure 4b. This crystallization likely induces phase separation, disrupting the uniformity of the polymer network and resulting in localized crystalline aggregates. The mean apparent densities (ρ_app_) of HPMC, HPMC-CTAB5, HPMC-CPC20, and HPMC-CTAC20 cryogels amounted to 0.034 ± 0.002 g/cm^3^, 0.038 ± 0.002 g/cm^3^, 0.040 ± 0.003 g/cm^3^, and 0.042 ± 0.002 g/cm^3^, respectively. These values are similar to other density values reported for HPMC-based cryogels^18,19^ and did not indicate any substantial increase due to the presence of surfactants.^18,19^

**Figure 5:**
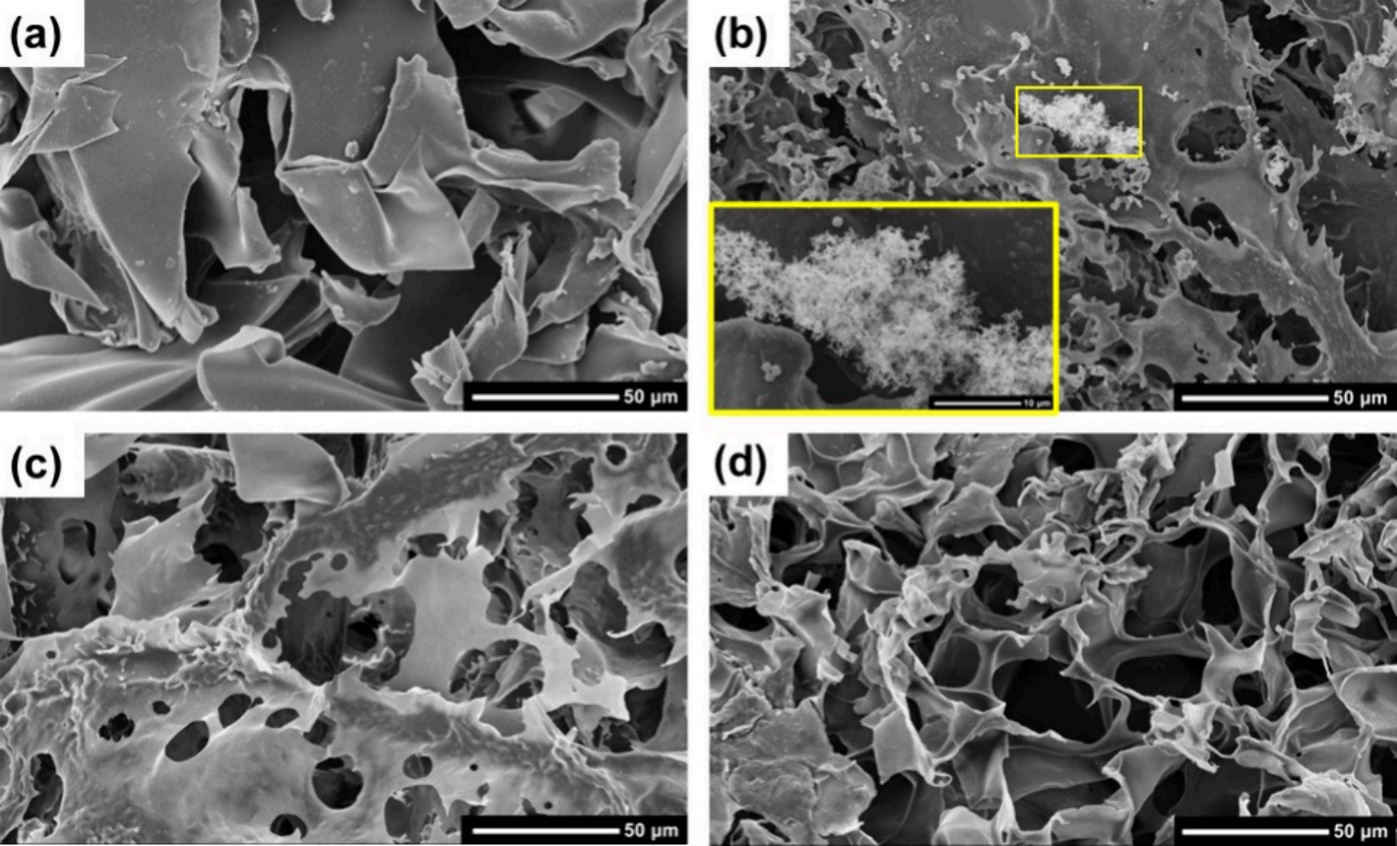
SEM images of (a) pure HPMC, (b) HPMC-CTAB5, (c) HPMC-CTAC20, and (d) HPMC-CPC20 cryogels. The scale bars correspond to 50 μm. The scale bar in the inset in (b) corresponds to 10 μm.

Figure S10 shows the typical stress–strain curves obtained for HPMC, HPMC-CTAB5, HPMC-CTAC20, and HPMC-CPC20 cryogels. Each sample showed an elastic region up to ∼15% strain, followed by plastic deformation. However, HPMC-CTAB5 was less compressible than the other compositions. The mean Young’s modulus (*E*) values determined for HPMC, HPMC-CTAB5, HPMC-CPC20, and HPMC-CTAC20 cryogels amounted to 228 ± 20 kPa, 11.6 ± 0.9 kPa, 176 ± 24 kPa, and 603 ± 96 kPa, respectively. Although there was no significant difference in the ρ_app_ values, the *E* values followed the order HPMC-CTAC20 > HPMC > HPMC-CPC20 > HPMC-CTAB5. As observed in the precursor hydrogels, the charged headgroups of CTAC micelles act as junction points among the HPMC chains, which remained after freeze-drying, making them the stiffest system. HPMC cryogels were slightly stiffer than HPMC–CPC (*p* = 0.013), indicating that the CPC pyridinium headgroups are more screened than the CTAC quaternary ammonium headgroups.

Figure 6 presents the storage moduli (*G*′) of precursor HPMC, HPMC-CTAC20, HPMC-CTAB20, and HPMC-CPC20 hydrogels at 1 and 10 Hz plotted against the elastic modulus (*E*) values of the corresponding cryogels. As expected, the *G*′ values at 1 Hz were lower than those at 10 Hz. At low frequency (1 Hz), the rate of deformation is slow relative to the dynamics of the network, allowing physical entanglements and junction points to form and dissipate more readily. In contrast, at high frequency (10 Hz), the polymer chains and micellar structures cannot reorganize within the oscillation time scale, resulting in more persistent entanglements and junctions. This restriction in chain mobility leads to higher *G*′ values. The *G*′ values at both 1 and 10 Hz increased exponentially with the corresponding *E* values. A similar trend was observed for systems composed of HPMC and SDS micelles.^18^ In such systems, the charged headgroups of the micelles act as junction points between HPMC chain segments, thereby reinforcing hydrogels and cryogels. However, when these headgroups are electrostatically screened, as in the case of HPMC–CPC, or when the micelles begin to crystallize, as observed in HPMC–CTAB systems, their ability to adhere to the polymer chains diminishes, resulting in softer hydrogel and cryogel structures.

**Figure 6:**
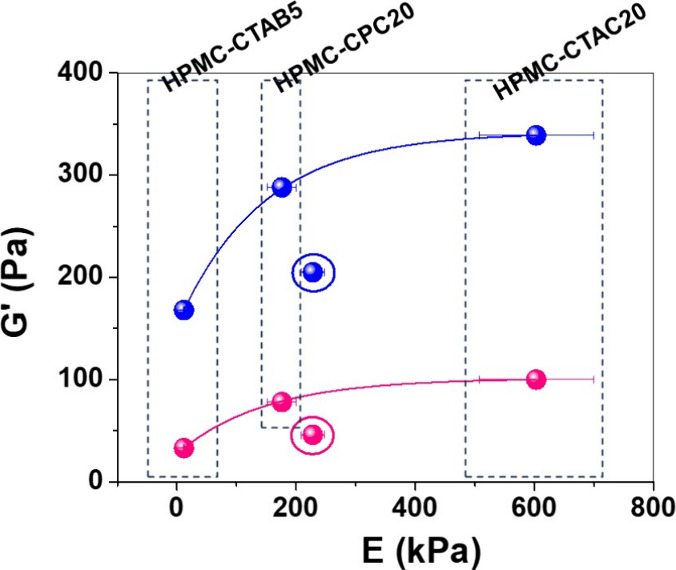
*G*′ values at 1 (pink symbols) and 10 Hz (blue symbols) of hydrogels as a function of *E* values determined for the corresponding cryogels. The circles indicate the *G*′ and *E* moduli of pure HPMC.

## 4 Conclusions

The rheological evaluation of HPMC hydrogels containing CTAB, CTAC, or CPC revealed that both the surfactant headgroup and the degree of counterion dissociation significantly influenced their mechanical properties. Among the systems studied, the addition of CTAC resulted in the most pronounced decrease in both the crossover modulus (where *G*′ = *G*″) and the corresponding frequency. This behavior indicates strong multipoint attachment between CTA^+^ micellar surfaces and HPMC chains, facilitated by the high degree of dissociation of the chloride ions. The elevated surface charge density of CTA^+^ micelles increased the electrostatic repulsion between them, leading to greater intermicellar spacing, as confirmed by the SAXS measurements. These multiple favorable interactions between micelles and polymer chains yielded cryogels with the highest Young’s modulus (∼600 kPa). In contrast, the flat structure of the pyridinium headgroup in CPC promotes stronger association with chloride ions, resulting in lower ion dissociation compared to CTAC. Consequently, the stiffening effect on both hydrogels and cryogels was less pronounced. For CTAB, the weak dissociation of bromide ions hindered strong electrostatic interactions between micelles and HPMC, resulting in fewer effective junction points in the hydrogel network. Additionally, the tendency of CTAB micelles to crystallize during the freezing process further diminished their interaction with HPMC, producing fragile cryogels.

These findings clearly demonstrate that the mechanical properties of HPMC-based hydrogels and cryogels can be finely tuned through the selection of surfactants with different headgroups and counterion dissociation behaviors, without altering the polymer concentration.

## Supplementary Material



## Notes

Dynamic frequency sweep tests were performed at 0.5% strain (Figure S1), typical SAXS curves obtained for CTAB, CTAC, and CPC aqueous solutions at 20 mM, along with the best fitting curves (Figure S2), structural parameters extracted from SAXS fitting curves (Figure S3), viscosity flow curves for pure HPMC (30 g/L), HPMC-CTAB20, HPMC-CTAC20, and HPMC-CPC20 hydrogels at 25 °C (Figure S4), Arrhenius plot for pure HPMC hydrogel (Figure S5), Arrhenius plot for HPMC-CTAC20 hydrogel (Figure S6), temperature values corresponding to the maximum (*T*max) and minimum (*T*min) indicated as points (i) and (ii), indicated in Figure 4b (Figure S7), viscosity (η) measurements performed at a constant shear rate of 1 s^–1^ under a cooling–heating cycle (Figure S8), FTIR-ATR spectra of HPMC, HPMC-CTAB20, HPMC-CTAC20, and HPMC-CPC20 cryogels (Figure S9), typical stress–strain curves obtained for HPMC, HPMC-CTAB5, HPMC-CTAC20, and HPMC-CPC20 cryogels (Figure S10) (PDF)
