# Supplementary material for: Structure–Property Relationship in Cationic Surfactant/Hydroxypropyl Methylcellulose Hydrogels and Cryogels: Role of Headgroup and Counterion Dissociation
Source: ACS Omega. 2025 Nov 12;10(46):56120–9. doi: 10.1021/acsomega.5c07745 (PMC12658781; doi:10.1021/acsomega.5c07745)

Structure–property relationship in cationic surfactant/hydroxypropyl methylcellulose hydrogels and cryogels: Role of head group and counter-ion dissociation

Victor B. Astuto[1], Rodrigo Fernandes[2,3], Rosangela Itri[2], Denise F. S. Petri[1]*

[1] Fundamental Chemistry Department, Institute of Chemistry, University of São Paulo, Av. Prof. Lineu Prestes 748, 05508-000 São Paulo, Brazil; vastuto@iq.usp.br, dfsp@iq.usp.br*

[2] Institute of Physics, University of São Paulo, São Paulo 05508-090, Brazil; rodrigo.fernandes.me@usp.br, itri@if.usp.br

[3] Department of Life and Environmental Sciences, Università Politecnica delle Marche, via Brecce Bianche, 60131 Ancona, Italy.

Figure S1 - Dynamic frequency sweep tests for pure HPMC (30 g/L), HPMC-CTAB20, HPMC-CTAC20, and HPMC-CPC20.

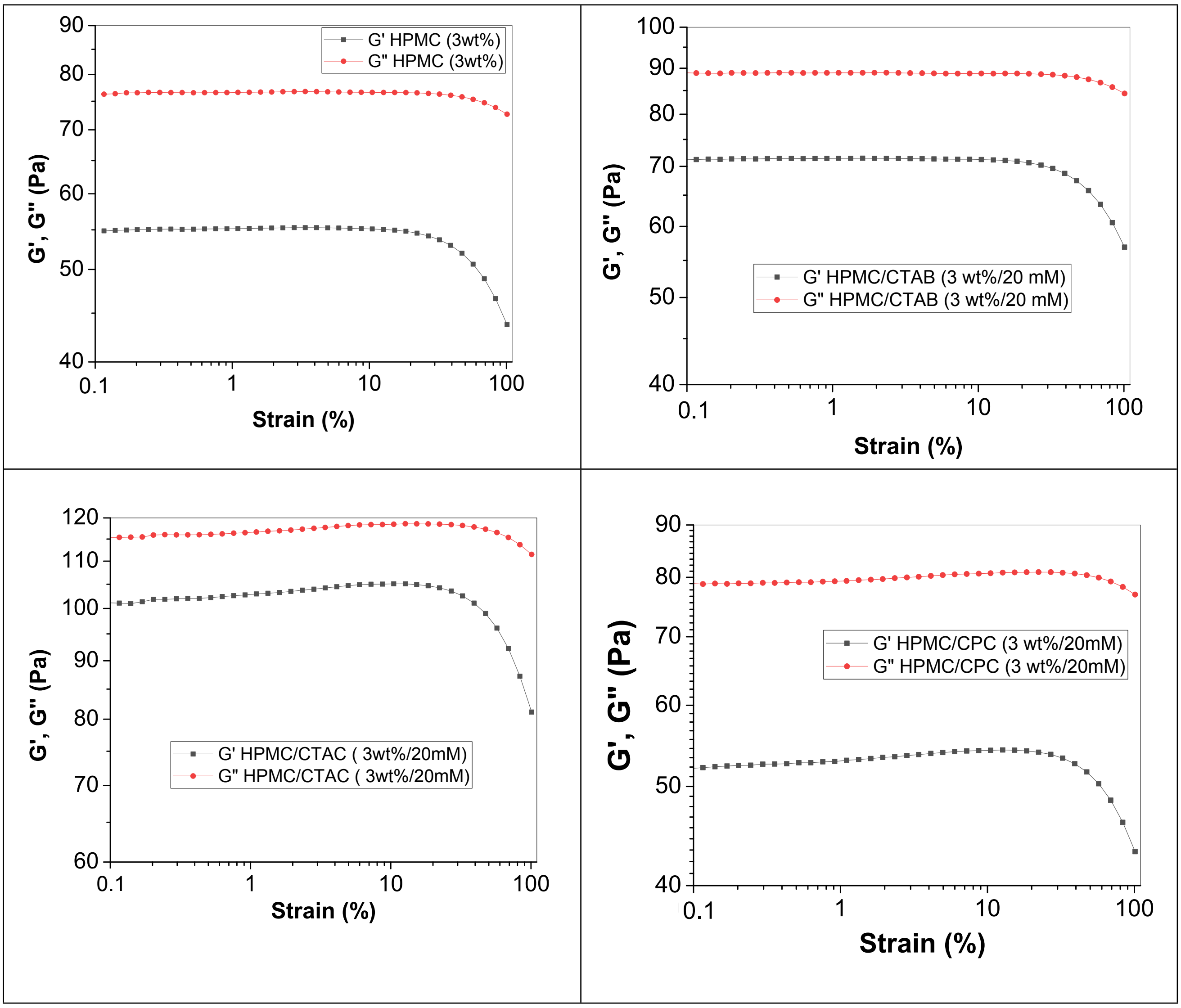

Figure S2 - Experimental SAXS data determined for CTAB, CTAC, and CPC aqueous solutions at 20 mM along with the fitting curves (red lines).

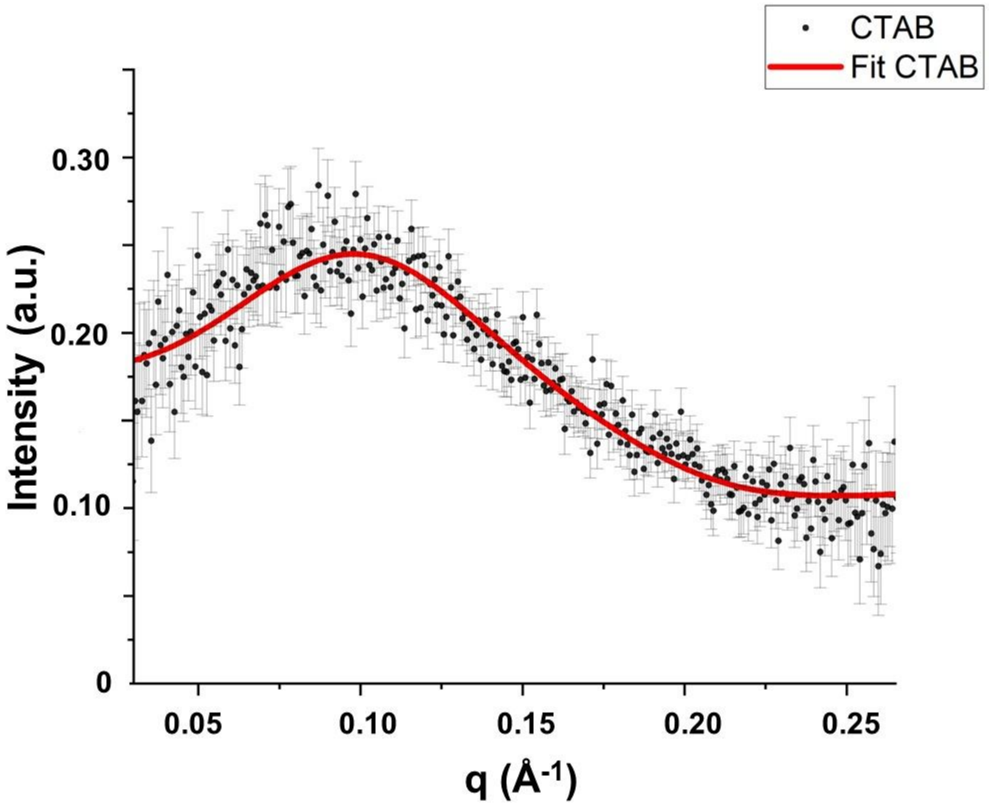

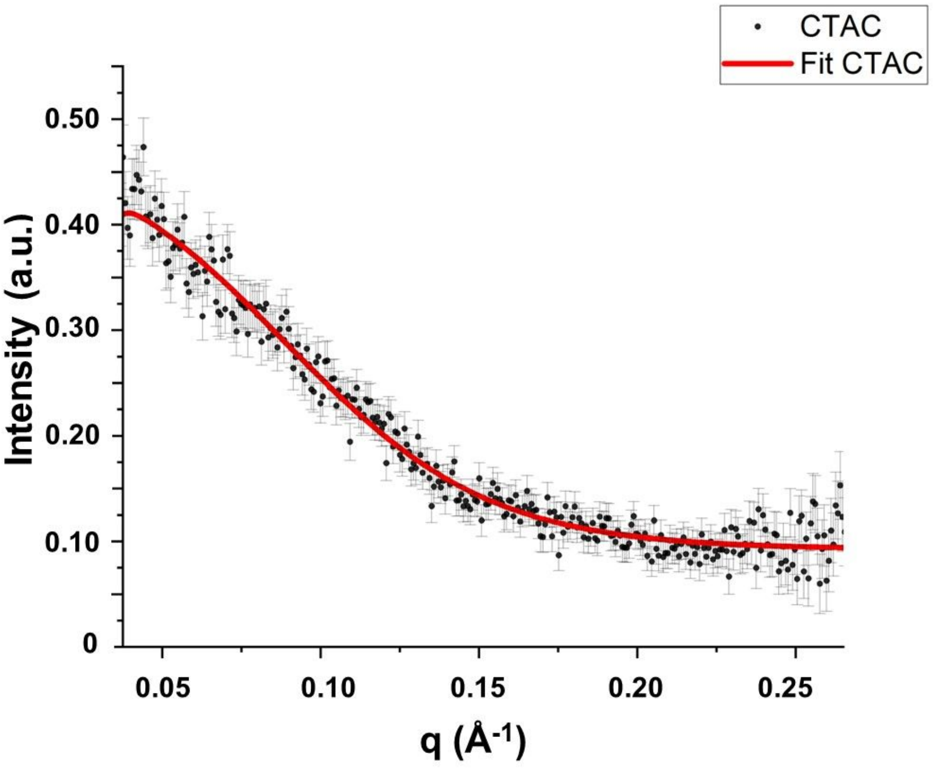

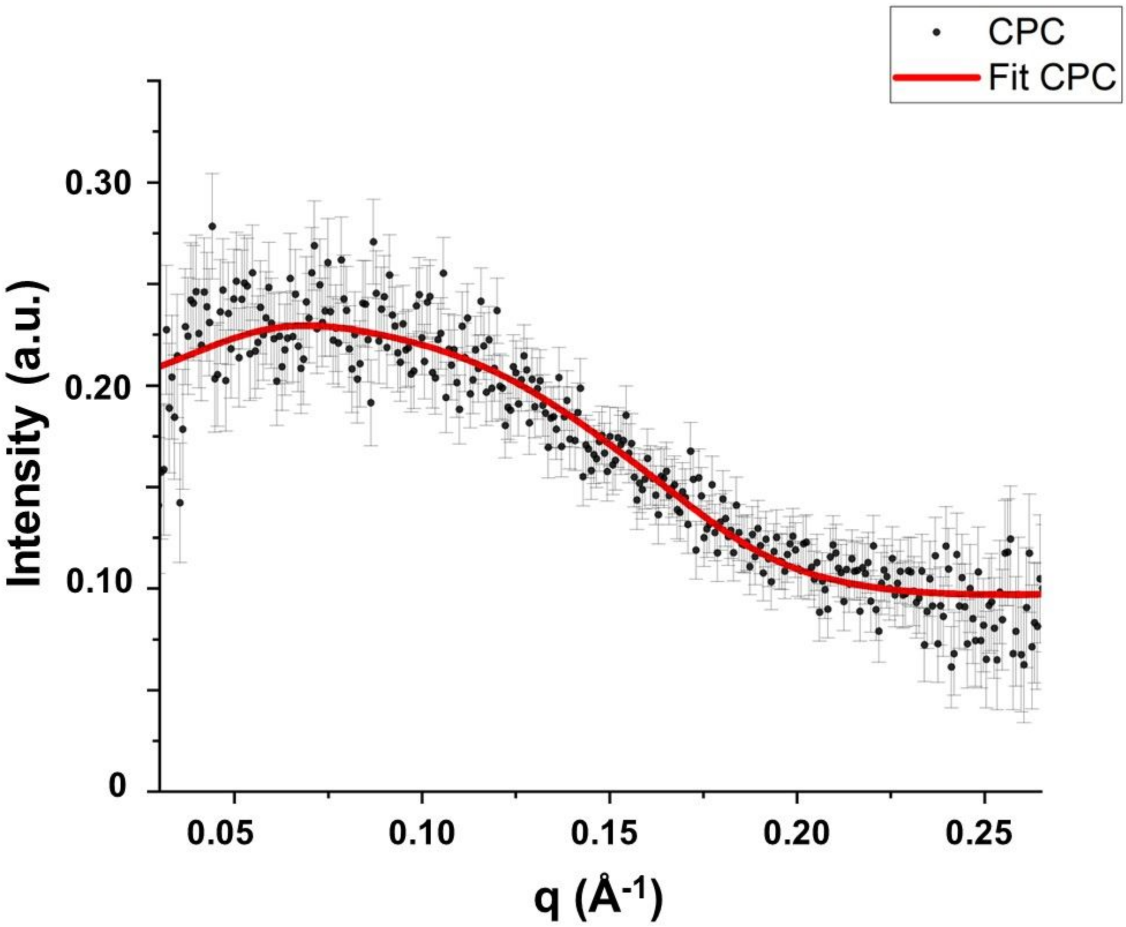

CPC
Fit CPC
Intensity (a.u.)
0
0.10
0.20
0.30
0.05
0.10
0.15
0.20
0.25
q (Å-1)

Figure S3. Structural parameters determined from SAXS measurements for CTAB, CTAC, and CPC micelles in water, at (25 ± 1) °C. Anisometry (ν), r_{par} (paraffinic radius), σ (thickness of the polar shell), electron density of the shell (ρ_{shell}), the electron density of the hydrophobic paraffinic core was set as ρ_{par} = 0.275 e/Å^3. Literature data [33-35] were presented for comparison.

System	ν	r_{par} (Å)	σ (Å)	ρ_{shell} (e/Å^3)	Reference
**$CTAB_{exp}$**	2.5	22.7 ± 2.2	5.2 ± 1.4	0.35 ± 0.03	This work
**$CTAB_{calc}$**	-	22.4	5.8	-	[33]
**$CTAC_{exp}$**	3.2	25.8 ± 4.4	5.1 ± 1.3	0.343 ± 0.004	This work
**$CTAC_{calc}$**	-	25.5	3.47	-	[34]
**CPC_{exp}**	2.5	20.8 ± 2.4	5.3 ± 1.6	0.35 ± 0.01	This work
**$CPC_{exp\text{-}DLS}$**	2.4	22.1	9.0	-	[35]

Figure S4 - Viscosity (η) flow curves determined for pure HPMC (30 g/L), HPMC-CTAB20, HPMC-CTAC20, and HPMC/CPC20 hydrogels at 25 °C.

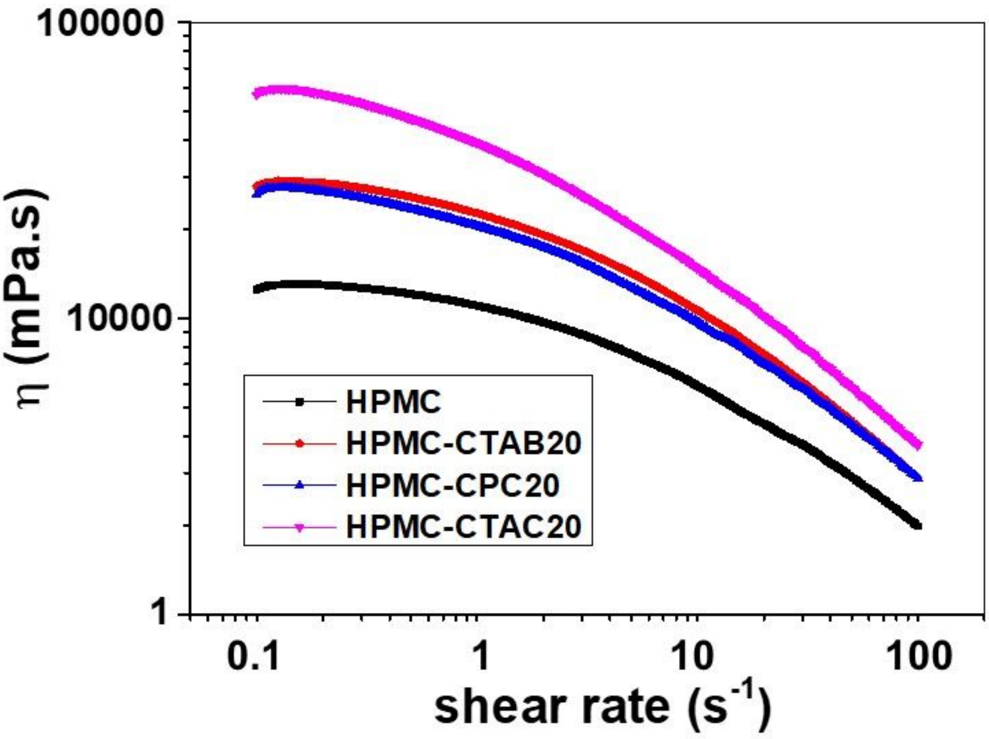

Fitting parameters to Carreau model: **A1** stands for zero shear viscosity and **n** stands for power index, when n = 1.0 the fluid has Newtonian behavior.

Sample	A1 (mPa.s)	n
HPMC	13525	0.4968
HPMC + 20 mM CPC	31441	0.3902
HPMC + 20 mM CTAB	31586	0.3736
HPMC + 20 mM CTAC	68123	0.4270

Figure S5 – Arrhenius plot for pure HPMC hydrogel and the corresponding linear fitting (red line), $R^2 = 0.9998$. The slope m is:

$$m = \frac{Ea}{R}$$

where Ea is the flow activation energy and R is the gas constant ($8.314\ J.K^{-1}.mol^{-1}$).

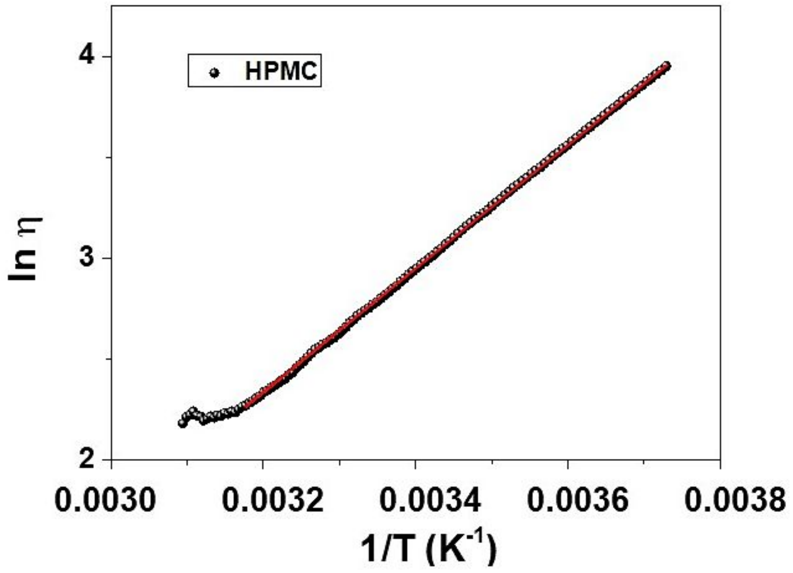

From the linear fitting, m = 3079.55 K. The Ea value was calculated as 25603 J mol^{-1}.

Figure S6 – Arrhenius plot for HPMC-CTAC20 hydrogel and the corresponding linear fitting (red line), $R^2 = 0.9755$. The slope m was 1080.91 K and the Ea value was calculated as 8987 J mol^{-1}.

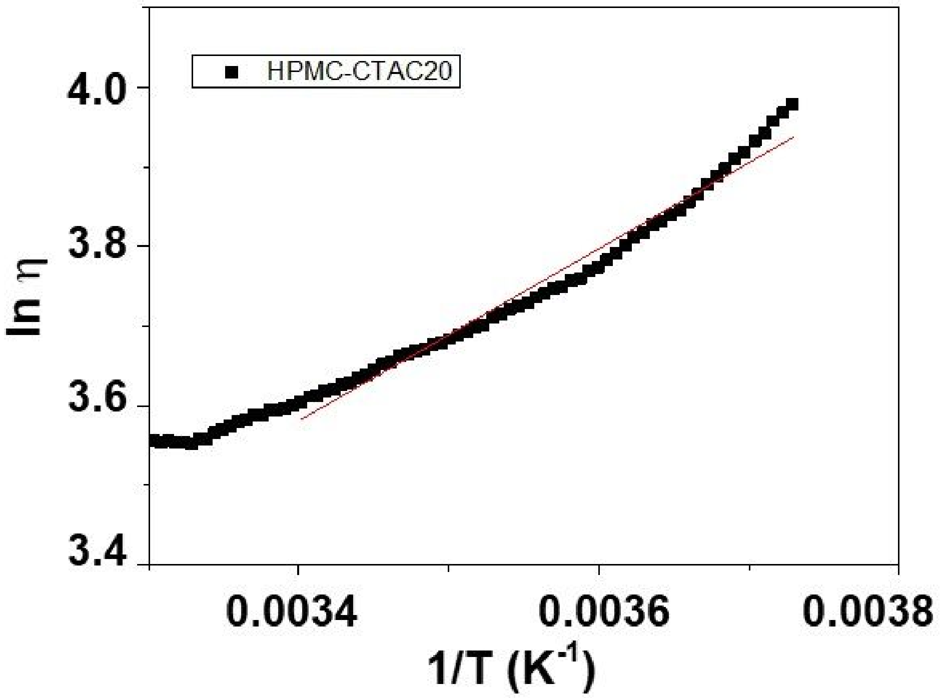

Figure S7. Temperature values corresponding to the maximum (Tmax) and minimum (Tmin) indicated as points (i) and (ii), indicated in Figure 4b, as a function of the CTAB concentration in the HPMC hydrogels.

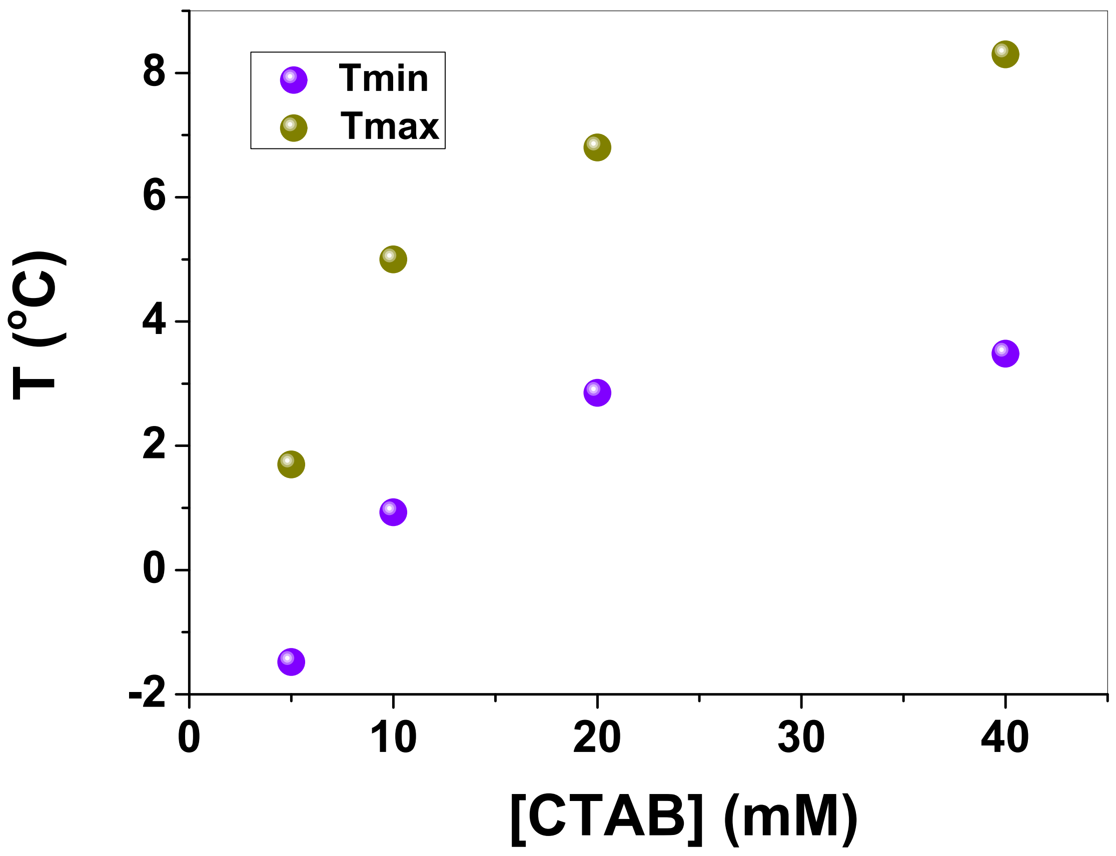

Figure S8. Viscosity (η) measurements performed at a constant shear rate of 1 s^{-1}, under cooling/heating cycle at a controlled cooling ramp from +50 °C to –5 °C, and at a rate of 1 °C/min: (a) HPMC (30 g/L), (b) HPMC-CTAB5, (c) HPMC-CTAB20, and (d) HPMC-CTAB40 hydrogels.

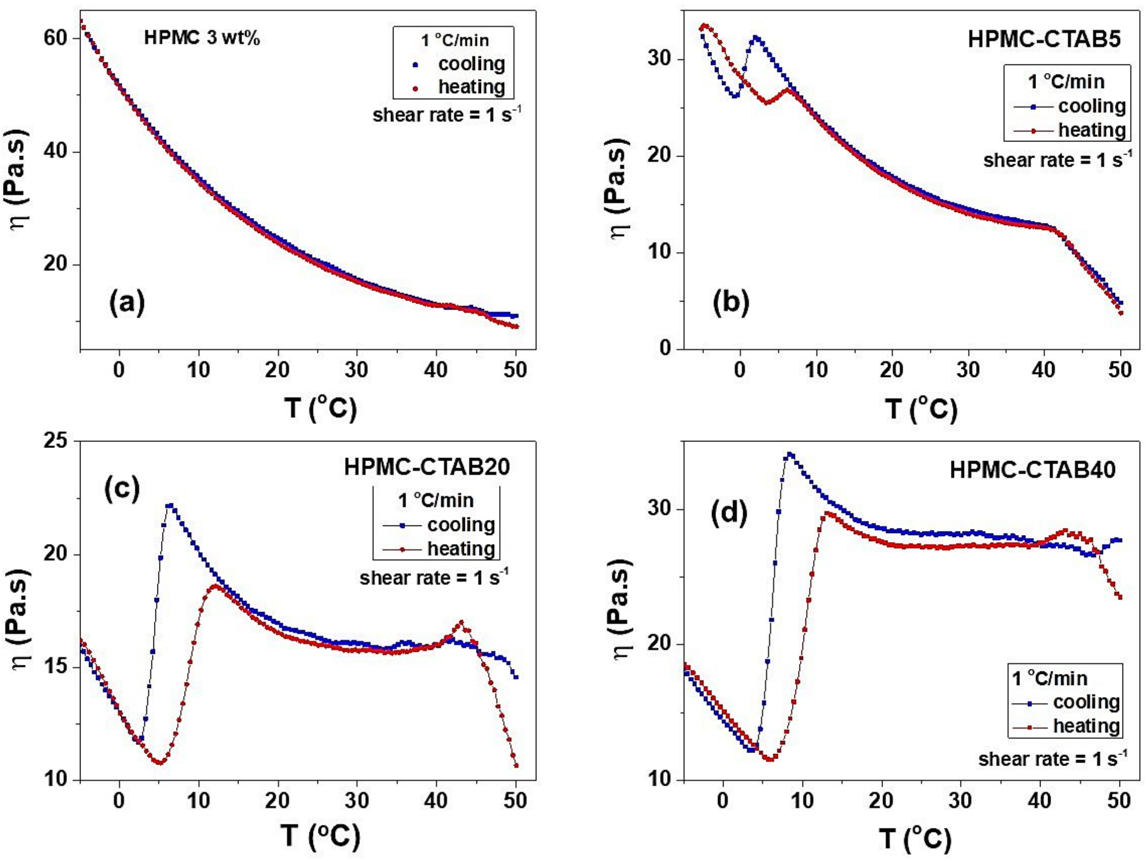

Figure S9. FTIR-ATR spectra obtained for HPMC, HPMC-CTAB20, HPMC-CTAC20, and HPMC-CPC20 cryogels.

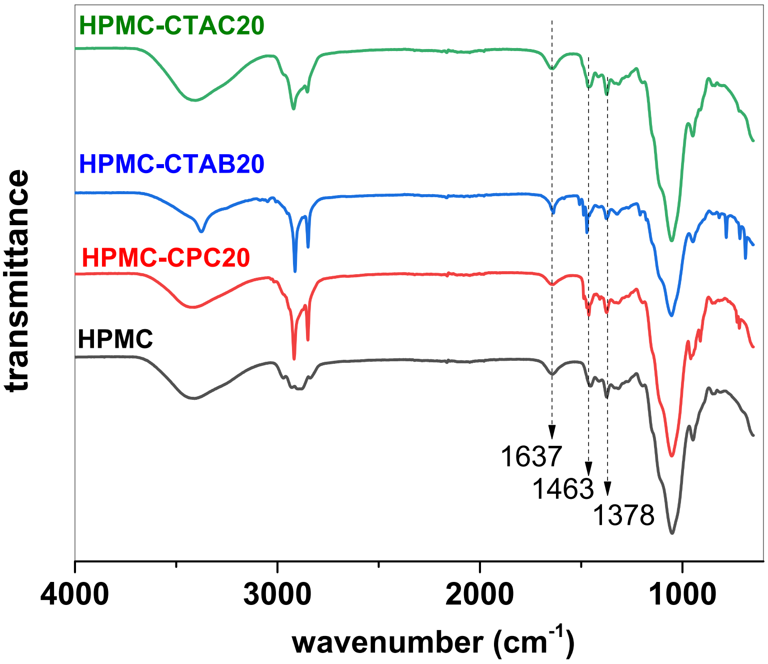

Figure S10. Typical stress-strain curves obtained for HPMC, HPMC-CTAB5, HPMC-CTAC20, and HPMC-CPC20 cryogels.

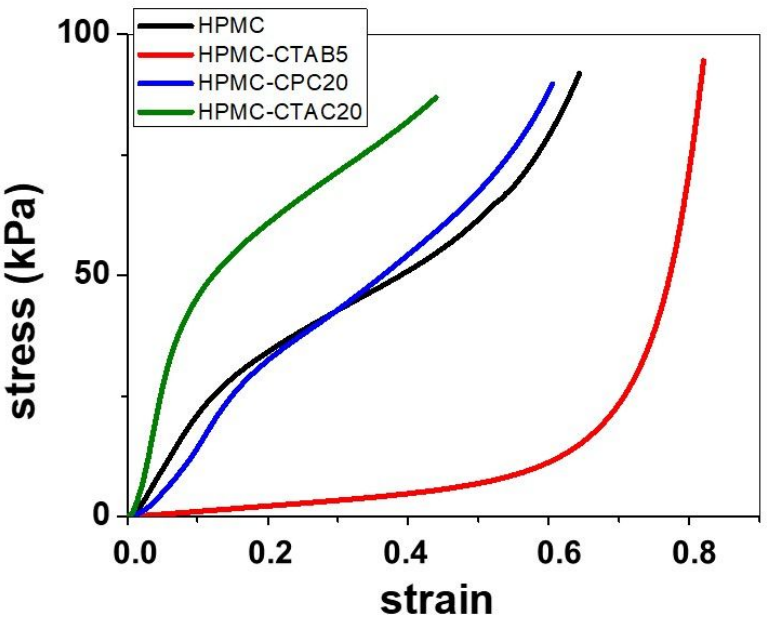

Supplement: Supplementary file 1 [file ao5c07745_si_001.pdf]
